# The genome sequence of the Vapourer moth, *Orgyia antiqua* (Linnaeus, 1758)

**DOI:** 10.12688/wellcomeopenres.19480.2

**Published:** 2024-10-15

**Authors:** Jamie C. Weir, University of Oxford and Wytham Woods Genome Acquisition Lab, Darwin Tree of Life Barcoding collective, Wellcome Sanger Institute Tree of Life programme, Wellcome Sanger Institute Scientific Operations: DNA Pipelines collective, Tree of Life Core Informatics collective, Darwin Tree of Life Consortium, Douglas Boyes

**Affiliations:** 1 Institute for Ecology and Evolution, The University of Edinburgh, Edinburgh, Scotland, UK; 2 UK Centre for Ecology & Hydrology, Wallingford, England, UK

**Keywords:** Orgyia antiqua, Vapourer moth, genome sequence, chromosomal, Lepidoptera

## Abstract

We present a genome assembly from an individual male *Orgyia antiqua* specimen (the Vapourer moth; Arthropoda; Insecta; Lepidoptera; Erebidae). The genome sequence is 480.1 megabases in span. Most of the assembly is scaffolded into 14 chromosomal pseudomolecules, including the Z sex chromosome. The mitochondrial genome has also been assembled and is 15.4 kilobases in length. Gene annotation of this assembly on Ensembl identified 12,475 protein coding genes.

## Species taxonomy

Eukaryota; Metazoa; Ecdysozoa; Arthropoda; Hexapoda; Insecta; Pterygota; Neoptera; Endopterygota; Lepidoptera; Glossata; Ditrysia; Noctuoidea; Erebidae; Lymantriinae; *Orgyia*; *Orgyia antiqua* (Linnaeus, 1758) (NCBI:txid335469).

## Background

**Figure 1.**
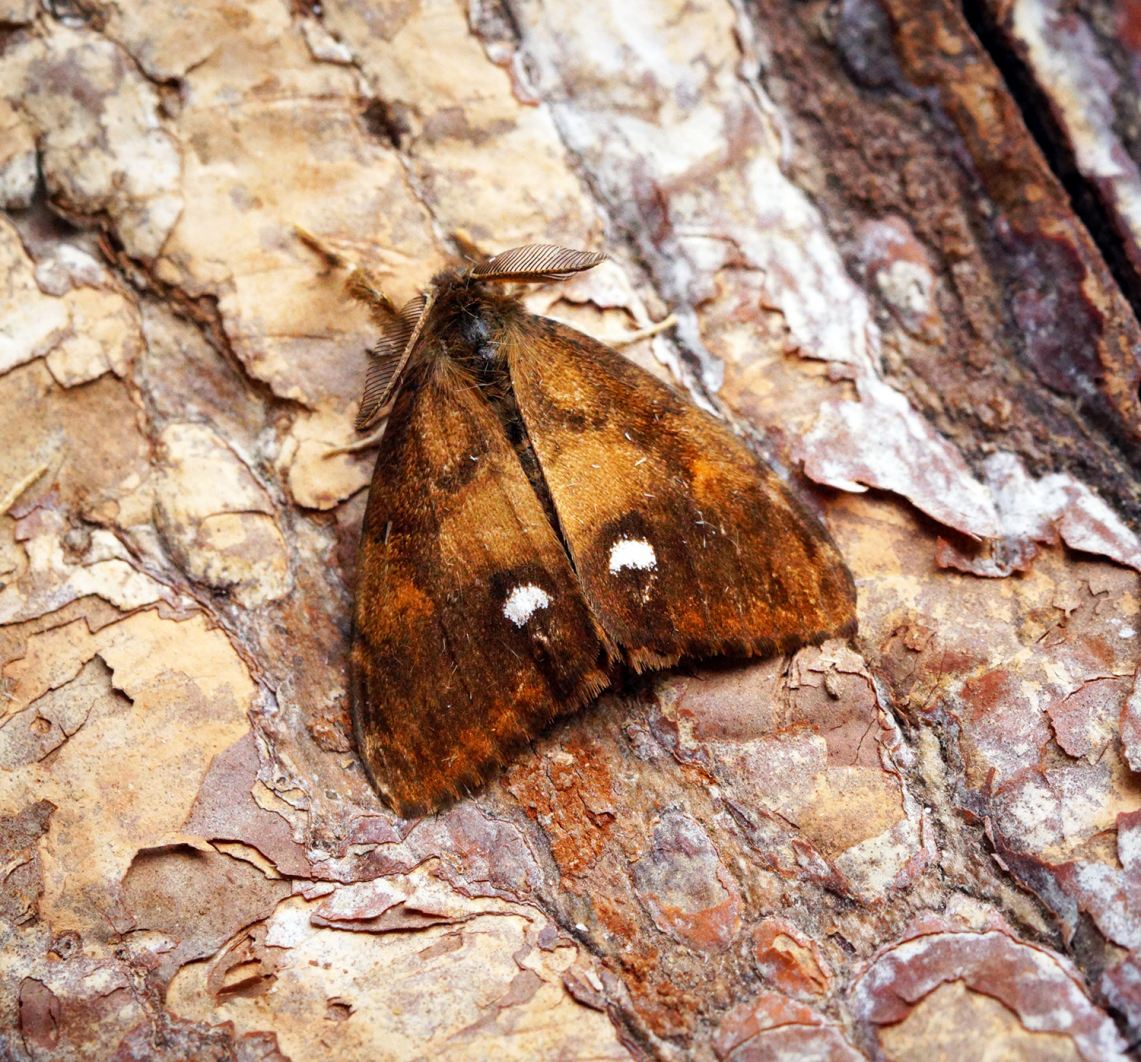
Photograph of *Orgyia antiqua* © Ben Sale.

The Vapourer or Rusty Tussock Moth *Orgyia antiqua* ( Figure 1) is a common lymantrin moth native to the European Palaearctic, but which has expanded into North America ( GBIF Secretariat, 2022). It is widespread throughout a range of habitats wherever usable host plants are present, from parks and gardens to woodlands and scrublands ( Waring *et al.*, 2017).

Adults are facultatively polyvoltine, producing several broods where environmental conditions are favourable. In Britain, however, there appears to be a single brood with a protracted emergence period across the summer ( Waring *et al.*, 2017), but *cf*. ( Emmet & Heath, 1983; Skinner & Wilson, 2009). Adult Vapourer moths are sexually dimorphic in both colouration and morphology. They do not feed as adults and are therefore capital breeders ( Tammaru & Haukioja, 1996) whose energetic requirements as an adult are met entirely by the resources they were able to gather as a caterpillar.

Females are brachypterous or micropterous ( Sattler, 1991), incapable of flight, and far larger than the males, having been reduced to simple egg-laying machines – indeed, evidence seems to suggests that ever larger body sizes, with larger egg complements, are consistently favoured in the wild ( Tammaru *et al.*, 2002). Males are fully winged, and fly by both day and night seeking out females using pheromone signals ( Waring *et al.*, 2017). In their non-native range, male Vapourer moths have been recorded at pheromone lures for other local *Orgyia* species.

Females often spend the entirety of their brief lives in or on their old cocoon ( Wickman *et al.*, 1975), where they lay large egg batches ( Emmet & Heath, 1983). Females may be capable of parthenogenetic reproduction ( Cockayne, 1938), and hybridisation can occur readily in captivity with other *Orgyia* species ( Robinson, 1971). Vapourer moths overwinter as ova ( Skinner & Wilson, 2009) and caterpillars hatch in early spring alongside the first flushes of foliage on their host plants ( Emmet & Heath, 1983; Waring *et al.*, 2017).

The caterpillars are highly polyphagous ( Robinson *et al.*, 2023), feeding on deciduous and coniferous trees, shrubs, and a range of low growing plants (e.g. *Vaccinium myrtillus*, *Plantago major*, *Bergenia crassifolia*). Both in their native and non-native range, vapourer caterpillars can reach outbreak densities and become a pest ( Emmet & Heath, 1983; Hewson & Mardon, 1970; Petersen, 1960). At the end of the larval period, which lasts for around 40–50 days, caterpillars pupate inside a neat, dome-like, silken cocoon, which incorporates scales and hairs from the caterpillar’s body, and eclose several weeks later ( Weir, 2022).

*Orygia antiqua* has been reported to have a karyotype of 14 chromosomes ( Cretschmar, 1928; Federley, 1932; Seiler, 1914) – summarised in Robinson (1971). Here we present a chromosomally complete genome sequence for *Orgyia antiqua*, sequences as part of the Darwin Tree of Life Project.

## Genome sequence report

The genome was sequenced from one male *Orgyia antiqua* collected from Airth, Scotland (latitude 56.07, longitude –3.77). A total of 54-fold coverage in Pacific Biosciences single-molecule HiFi long reads and 71-fold coverage in 10X Genomics read clouds was generated. Primary assembly contigs were scaffolded with chromosome conformation Hi-C data. Manual assembly curation corrected four missing joins or mis-joins, reducing the scaffold count by 4.

**Figure 2.**
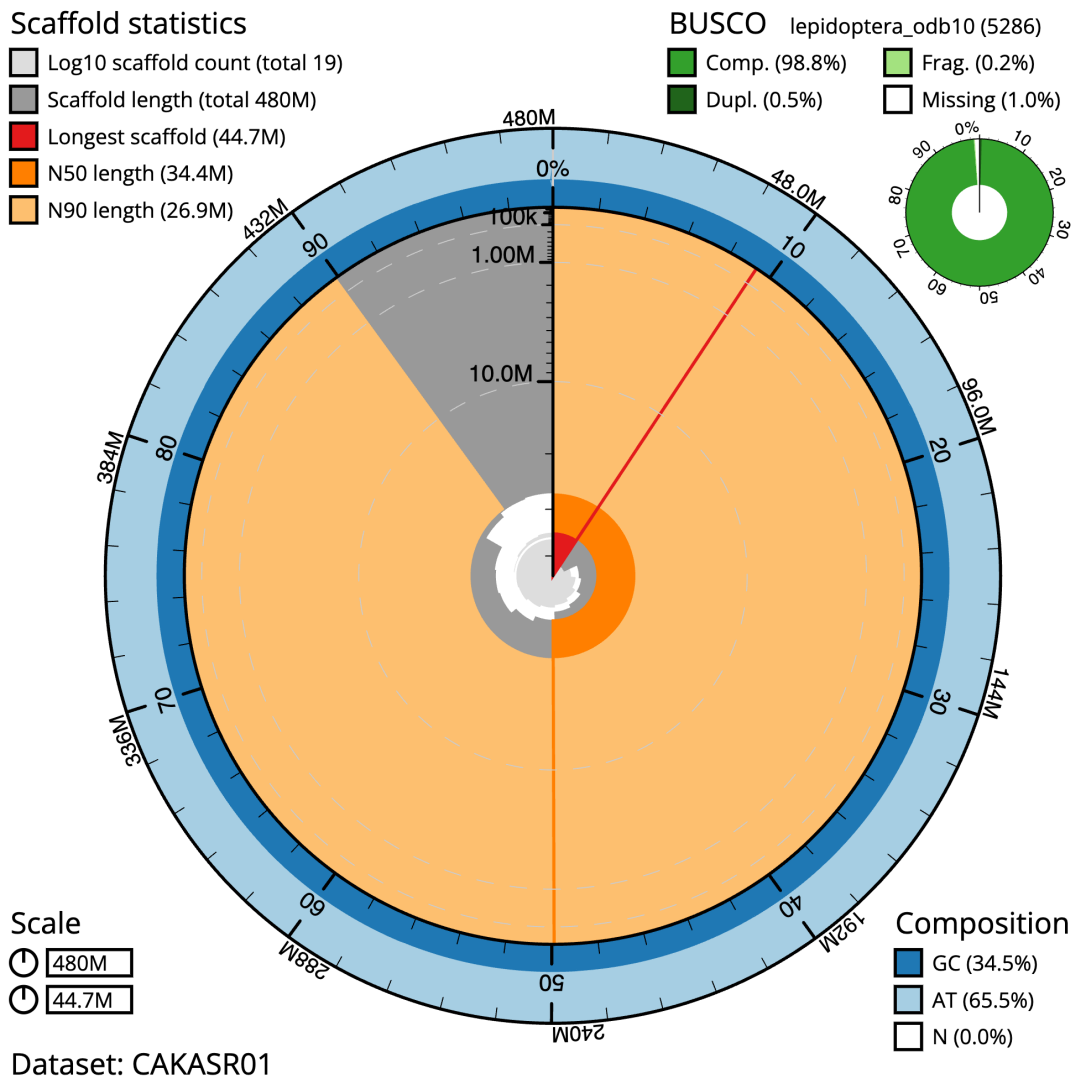
Genome assembly of *Orgyia antiqua*, ilOrgAnti1.1: metrics. The BlobToolKit Snailplot shows N50 metrics and BUSCO gene completeness. The main plot is divided into 1,000 size-ordered bins around the circumference with each bin representing 0.1% of the 480,110,421 bp assembly. The distribution of scaffold lengths is shown in dark grey with the plot radius scaled to the longest scaffold present in the assembly (44,675,926 bp, shown in red). Orange and pale-orange arcs show the N50 and N90 scaffold lengths (34,414,056 and 26,928,026 bp), respectively. The pale grey spiral shows the cumulative scaffold count on a log scale with white scale lines showing successive orders of magnitude. The blue and pale-blue area around the outside of the plot shows the distribution of GC, AT and N percentages in the same bins as the inner plot. A summary of complete, fragmented, duplicated and missing BUSCO genes in the lepidoptera_odb10 set is shown in the top right. An interactive version of this figure is available at https://blobtoolkit.genomehubs.org/view/Orgyia%20antiqua/dataset/CAKASR01/snail.

The final assembly has a total length of 480.1 Mb in 18 sequence scaffolds with a scaffold N50 of 34.4 Mb ( Table 1). Most (99.98%) of the assembly sequence was assigned to 14 chromosomal-level scaffolds, representing 13 autosomes and the Z sex chromosome. Chromosome-scale scaffolds confirmed by the Hi-C data are named in order of size ( Figure 2– Figure 5; Table 2). While not fully phased, the assembly deposited is of one haplotype. Contigs corresponding to the second haplotype have also been deposited. The mitochondrial genome was also assembled and can be found as a contig within the multifasta file of the genome submission.

**Table 1.** Genome data for *Orgyia antiqua*, ilOrgAnti1.1.

Project accession data		
Assembly identifier	ilOrgAnti1.1	
Species	*Orgyia antiqua*	
Specimen	ilOrgAnti1	
NCBI taxonomy ID	335469	
BioProject	PRJEB47377	
BioSample ID	SAMEA7524390	
Isolate information	ilOrgAnti1, male, whole organism (DNA sequencing and Hi-C scaffolding) ilOrgAnti2, whole organism (RNA sequencing)	
Assembly metrics *		*Benchmark*
Consensus quality (QV)	62.1	*≥ 50*
*k*-mer completeness	100%	*≥ 95%*
BUSCO **	C:98.8%[S:98.3%,D:0.5%], F:0.2%,M:1.0%,n:5,286	*C ≥ 95%*
Percentage of assembly mapped to chromosomes	99.98%	*≥ 95%*
Sex chromosomes	Z chromosome	*localised homologous pairs*
Organelles	Mitochondrial genome assembled	*complete single alleles*
Raw data accessions		
PacificBiosciences SEQUEL II	ERR6939280, ERR6939281, ERR6909088	
10X Genomics Illumina	ERR6688829–ERR6688832	
Hi-C Illumina	ERR6688833	
PolyA RNA-Seq Illumina	ERR9435025, ERR9435024	
Genome assembly		
Assembly accession	GCA_916999025.1	
*Accession of alternate haplotype*	GCA_917414775.1	
Span (Mb)	480.1	
Number of contigs	26	
Contig N50 length (Mb)	32.0	
Number of scaffolds	18	
Scaffold N50 length (Mb)	34.4	
Longest scaffold (Mb)	44.7	
Genome annotation		
Number of protein-coding genes	12,475	
Number of non-coding genes	2,486	
Number of gene transcripts	21,914	

* Assembly metric benchmarks are adapted from column VGP-2020 of “Table 1: Proposed standards and metrics for defining genome assembly quality” from ( Rhie *et al.*, 2021). ** BUSCO scores based on the lepidoptera_odb10 BUSCO set using v5.3.2. C = complete [S = single copy, D = duplicated], F = fragmented, M = missing, n = number of orthologues in comparison. A full set of BUSCO scores is available at https://blobtoolkit.genomehubs.org/view/Orgyia%20antiqua/dataset/CAKASR01/busco.

**Figure 3.**
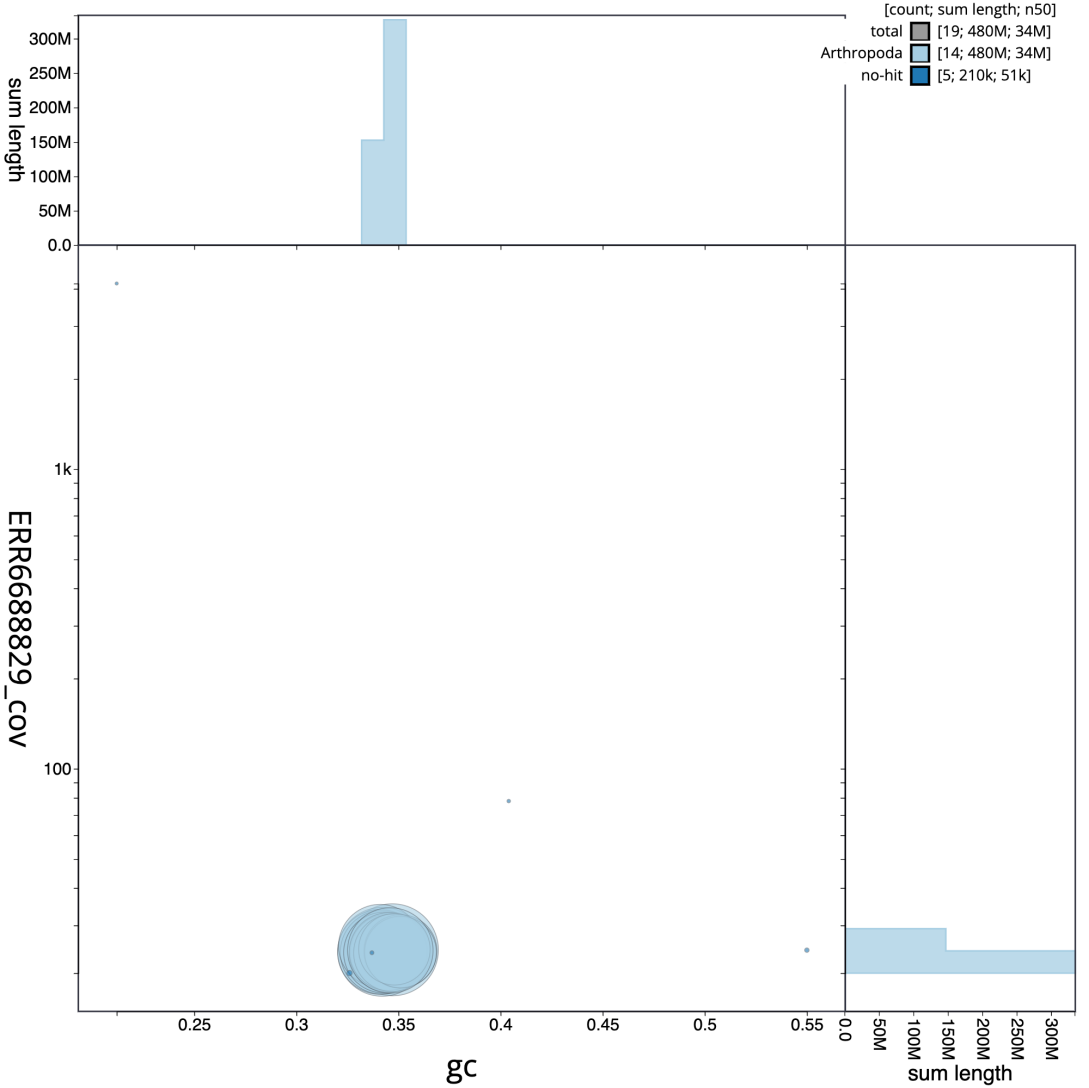
Genome assembly of *Orgyia antiqua*, ilOrgAnti1.1: BlobToolKit GC-coverage plot. Scaffolds are coloured by phylum. Circles are sized in proportion to scaffold length. Histograms show the distribution of scaffold length sum along each axis. An interactive version of this figure is available at https://blobtoolkit.genomehubs.org/view/Orgyia%20antiqua/dataset/CAKASR01/blob.

**Figure 4.**
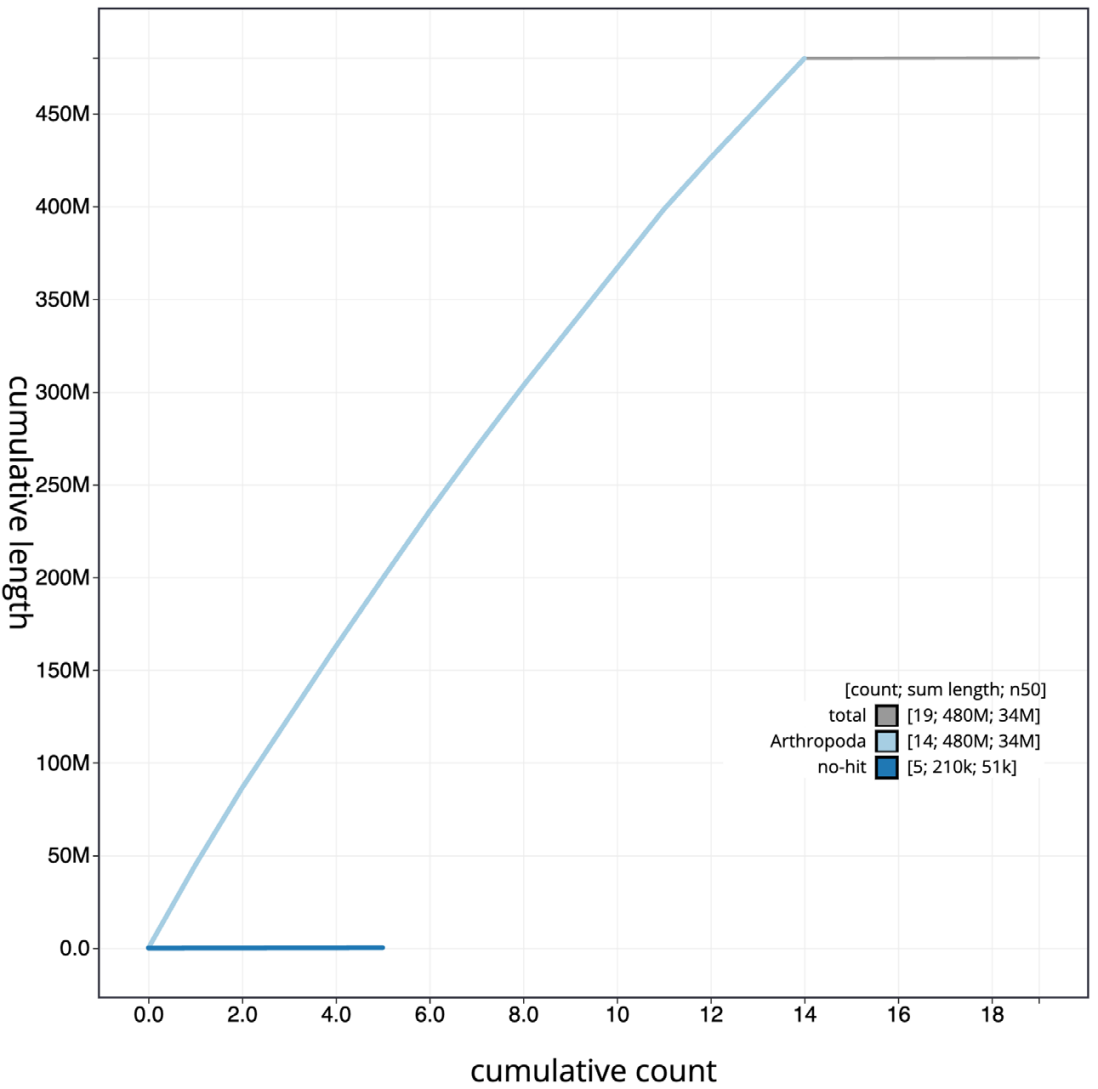
Genome assembly of *Orgyia antiqua*, ilOrgAnti1.1: BlobToolKit cumulative sequence plot. The grey line shows cumulative length for all scaffolds. Coloured lines show cumulative lengths of scaffolds assigned to each phylum using the buscogenes taxrule. An interactive version of this figure is available at https://blobtoolkit.genomehubs.org/view/Orgyia%20antiqua/dataset/CAKASR01/cumulative.

**Figure 5.**
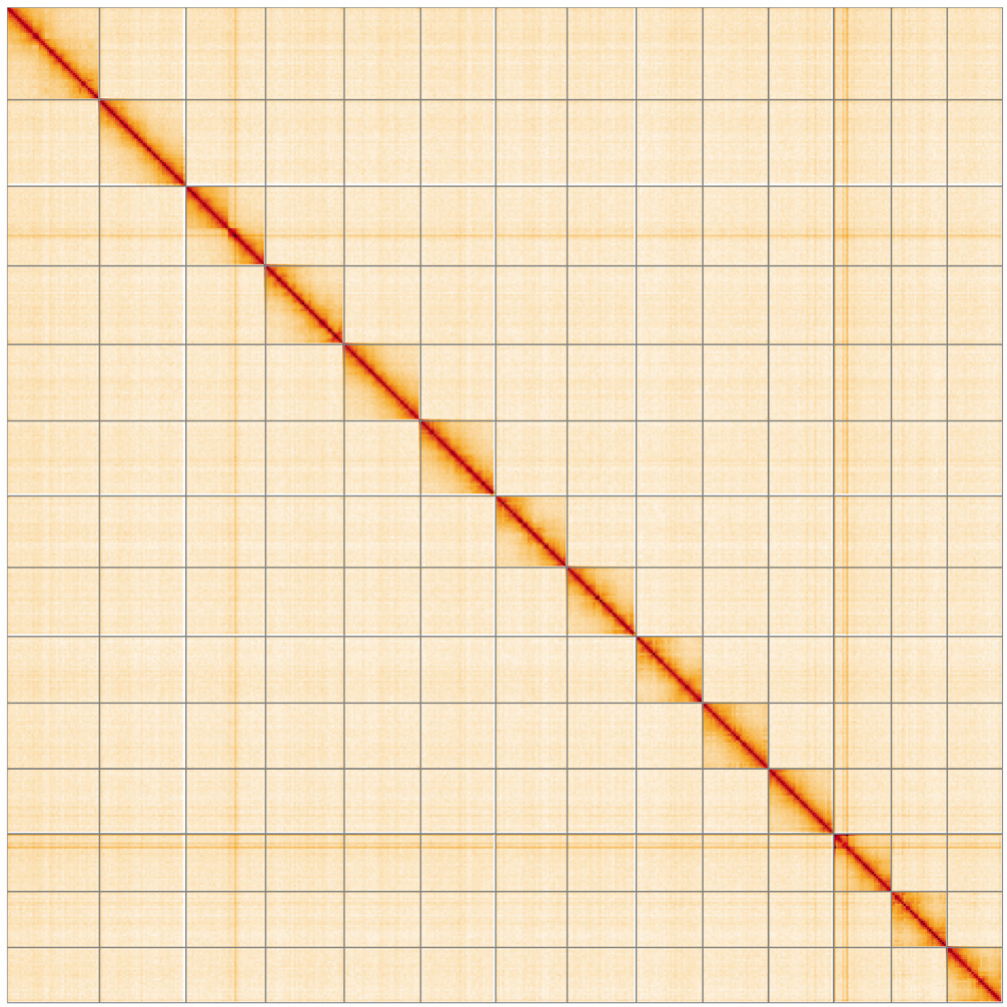
Genome assembly of *Orgyia antiqua*, ilOrgAnti1.1: Hi-C contact map of the ilOrgAnti1.1 assembly, visualised using HiGlass. Chromosomes are shown in order of size from left to right and top to bottom. An interactive version of this figure may be viewed at https://genome-note-higlass.tol.sanger.ac.uk/l/?d=HFeVSULIRt2W3gNX1egZ9g.

**Table 2.** Chromosomal pseudomolecules in the genome assembly of *Orgyia antiqua*, ilOrgAnti1.

INSDC accession	Chromosome	Size (Mb)	GC%
OU779861.1	1	44.68	34.7
OU779862.1	2	41.67	34.2
OU779864.1	3	37.85	34.1
OU779865.1	4	36.8	34.2
OU779866.1	5	36.18	34.1
OU779867.1	6	34.41	34.3
OU779868.1	7	33.3	34.6
OU779869.1	8	32	34.5
OU779870.1	9	31.7	34.5
OU779871.1	10	31.43	34.7
OU779872.1	11	27.85	35.1
OU779873.1	12	26.93	35.1
OU779874.1	13	26.71	34.8
OU779863.1	Z	38.4	34.6
OU779875.1	MT	0.02	21.3
-	unplaced	0.19	39.5

The estimated Quality Value (QV) of the final assembly is 62.1 with *k*-mer completeness of 100%, and the assembly has a BUSCO v5.3.2 completeness of 98.8% (single = 98.3%, duplicated = 0.5%), using the lepidoptera_odb10 reference set ( *n* = 5,286).

Metadata for specimens, spectral estimates, sequencing runs, contaminants and pre-curation assembly statistics can be found at https://links.tol.sanger.ac.uk/species/335469.

## Genome annotation report

The *Orgyia antiqua* genome assembly GCA_916999025.1 was annotated using the Ensembl rapid annotation pipeline ( Table 1; https://rapid.ensembl.org/Orgyia_antiqua_GCA_916999025.1/Info/Index). The resulting annotation includes 21,914 transcribed mRNAs from 12,475 protein-coding and 2,486 non-coding genes. The average transcript length is 14,793.51. There are 1.46 coding transcripts per gene and 6.75 exons per transcript.

## Methods

### Sample acquisition and nucleic acid extraction

Moth ova were collected from a garden in Airth, Scotland, UK (latitude 56.07, longitude –3.77) on 22 June 2020 by Jamie Weir (University of Edinburgh). A male *Orgyia antiqua* (ilOrgAnti1) specimen was reared *ex ova* to adulthood and was subsequently preserved on dry ice. This specimen was used for genome sequencing and Hi-C scaffolding.

A second *O. antiqua* specimen (specimen no. Ox000948, individual ilOrgAnti2) was collected by Douglas Boyes (University of Oxford) from Wytham Woods, Oxfordshire (biological vice-county: Berkshire), UK (latitude 51.77, longitude –1.34) on 8 September 2020, using a light trap. This individual was used for RNA sequencing.

The workflow for high molecular weight (HMW) DNA extraction at the Wellcome Sanger Institute (WSI) Tree of Life Core Laboratory includes a sequence of core procedures: sample preparation and homogenisation, DNA extraction, fragmentation and purification. Detailed protocols are available on protocols.io ( Denton *et al*., 2023b). The ilOrgAnti1 sample was weighed and dissected on dry ice with tissue set aside for Hi-C sequencing. Thorax tissue was disrupted using a Nippi Powermasher fitted with a BioMasher pestle ( Denton *et al*., 2023a). High molecular weight (HMW) DNA was extracted using the Qiagen MagAttract HMW DNA extraction kit ( Strickland *et al*., 2023b). Low molecular weight DNA was removed from a 20 ng aliquot of extracted DNA using the 0.8X AMpure XP purification kit prior to 10X Chromium sequencing; a minimum of 50 ng DNA was submitted for 10X sequencing. HMW DNA was sheared into an average fragment size of 12–20 kb in a Megaruptor 3 system with speed setting 30 ( Todorovic *et al*., 2023). Sheared DNA was purified by solid-phase reversible immobilisation using AMPure PB beads with a 1.8X ratio of beads to sample to remove the shorter fragments and concentrate the DNA sample ( Strickland *et al*., 2023a). The concentration of the sheared and purified DNA was assessed using a Nanodrop spectrophotometer and Qubit Fluorometer and Qubit dsDNA High Sensitivity Assay kit. Fragment size distribution was evaluated by running the sample on the FemtoPulse system.

RNA was extracted from abdomen tissue of ilOrgAnti2 in the Tree of Life Laboratory at the WSI using TRIzol, according to the manufacturer’s instructions. RNA was then eluted in 50 μl RNAse-free water and its concentration assessed using a Nanodrop spectrophotometer and Qubit Fluorometer using the Qubit RNA Broad-Range (BR) Assay kit. Analysis of the integrity of the RNA was done using Agilent RNA 6000 Pico Kit and Eukaryotic Total RNA assay.

### Hi-C preparation

Tissue from the head of the ilOrgAnti1 sample was processed at the WSI Scientific Operations core, using the Arima-HiC v2 kit. Tissue (stored at –80 °C) was fixed, and the DNA crosslinked using a TC buffer with 22% formaldehyde. After crosslinking, the tissue was homogenised using the Diagnocine Power Masher-II and BioMasher-II tubes and pestles. Following the kit manufacturer's instructions, crosslinked DNA was digested using a restriction enzyme master mix. The 5’-overhangs were then filled in and labelled with biotinylated nucleotides and proximally ligated. An overnight incubation was carried out for enzymes to digest remaining proteins and for crosslinks to reverse. A clean up was performed with SPRIselect beads prior to library preparation.

### Sequencing

Pacific Biosciences HiFi circular consensus and 10X Genomics read cloud DNA sequencing libraries were constructed according to the manufacturers’ instructions. Poly(A) RNA-Seq libraries were constructed using the NEB Ultra II RNA Library Prep kit. DNA and RNA sequencing were performed by the Scientific Operations core at the WSI on Pacific Biosciences SEQUEL II (HiFi), Illumina HiSeq 4000 (RNA-Seq) and HiSeq X Ten (10X) instruments. For Hi-C library preparation, DNA was fragmented to a size of 400 to 600 bp using a Covaris E220 sonicator. The DNA was then enriched, barcoded, and amplified using the NEBNext Ultra II DNA Library Prep Kit following manufacturers’ instructions. The Hi-C sequencing was performed using paired-end sequencing with a read length of 150 bp on an Illumina NovaSeq 6000 instrument.

### Genome assembly, curation and evaluation

Assembly was carried out with Hifiasm with the --primary option ( Cheng *et al.*, 2021) and haplotypic duplication was identified and removed with purge_dups ( Guan *et al.*, 2020). One round of polishing was performed by aligning 10X Genomics read data to the assembly with Long Ranger ALIGN, calling variants with FreeBayes ( Garrison & Marth, 2012). The assembly was then scaffolded with Hi-C data ( Rao *et al.*, 2014) using SALSA2 ( Ghurye *et al.*, 2019). The assembly was checked for contamination as described previously ( Howe *et al.*, 2021). Manual curation was performed using HiGlass ( Kerpedjiev *et al.*, 2018) and PretextView ( Harry, 2022). The mitochondrial genome was assembled using MitoHiFi ( Uliano-Silva *et al.*, 2022), which runs MitoFinder ( Allio *et al.*, 2020) and uses these annotations to select the final mitochondrial contig and to ensure the general quality of the sequence.

A Hi-C map for the final assembly was produced using bwa-mem2 ( Vasimuddin *et al.*, 2019) in the Cooler file format ( Abdennur & Mirny, 2020). To assess the assembly metrics, the *k*-mer completeness and QV consensus quality values were calculated in Merqury ( Rhie *et al.*, 2020). This work was done using Nextflow ( Di Tommaso *et al.*, 2017) DSL2 pipelines “sanger-tol/readmapping” ( Surana *et al.*, 2023a) and “sanger-tol/genomenote” ( Surana *et al.*, 2023b). The genome was analysed within the BlobToolKit environment ( Challis *et al.*, 2020) and BUSCO scores ( Manni *et al.*, 2021; Simão *et al.*, 2015) were calculated.

Table 3 contains a list of relevant software tool versions and sources.

**Table 3.** Software tools: versions and sources.

Software tool	Version	Source
BlobToolKit	4.0.7	https://github.com/blobtoolkit/blobtoolkit
BUSCO	5.3.2	https://gitlab.com/ezlab/busco
FreeBayes	1.3.1-17-gaa2ace8	https://github.com/freebayes/freebayes
Hifiasm	0.15.3	https://github.com/chhylp123/hifiasm
HiGlass	1.11.6	https://github.com/higlass/higlass
Long Ranger ALIGN	2.2.2	https://support.10xgenomics.com/genome-exome/ software/pipelines/latest/advanced/other-pipelines
Merqury	MerquryFK	https://github.com/thegenemyers/MERQURY.FK
MitoHiFi	2	https://github.com/marcelauliano/MitoHiFi
PretextView	0.2	https://github.com/wtsi-hpag/PretextView
purge_dups	1.2.3	https://github.com/dfguan/purge_dups
SALSA	2.2	https://github.com/salsa-rs/salsa
sanger-tol/genomenote	v1.0	https://github.com/sanger-tol/genomenote
sanger-tol/readmapping	1.1.0	https://github.com/sanger-tol/readmapping/tree/1.1.0

### Genome annotation

The Ensembl gene annotation system ( Aken *et al.*, 2016) was used to generate annotation for the *Orgyia antiqua* assembly (GCA_916999025.1). Annotation was created primarily through alignment of transcriptomic data to the genome, with gap filling via protein-to-genome alignments of a select set of proteins from UniProt ( UniProt Consortium, 2019).

### Legal and ethical review process for Darwin Tree of Life Partner submitted materials

The materials that have contributed to this genome note have been supplied by a Darwin Tree of Life Partner.

The submission of materials by a Darwin Tree of Life Partner is subject to the **‘Darwin Tree of Life Project Sampling Code of Practice’**, which can be found in full on the Darwin Tree of Life website here. By agreeing with and signing up to the Sampling Code of Practice, the Darwin Tree of Life Partner agrees they will meet the legal and ethical requirements and standards set out within this document in respect of all samples acquired for, and supplied to, the Darwin Tree of Life Project.

Further, the Wellcome Sanger Institute employs a process whereby due diligence is carried out proportionate to the nature of the materials themselves, and the circumstances under which they have been/are to be collected and provided for use. The purpose of this is to address and mitigate any potential legal and/or ethical implications of receipt and use of the materials as part of the research project, and to ensure that in doing so we align with best practice wherever possible.

The overarching areas of consideration are:

- Ethical review of provenance and sourcing of the material
- Legality of collection, transfer and use (national and international)

Each transfer of samples is further undertaken according to a Research Collaboration Agreement or Material Transfer Agreement entered into by the Darwin Tree of Life Partner, Genome Research Limited (operating as the Wellcome Sanger Institute), and in some circumstances other Darwin Tree of Life collaborators.

## Data Availability

European Nucleotide Archive: *Orgyia antiqua* (rusty tussock moth). Accession number PRJEB47377; https://identifiers.org/ena.embl/PRJEB47377. ( Wellcome Sanger Institute, 2021) The genome sequence is released openly for reuse. The *Orgyia antiqua* genome sequencing initiative is part of the Darwin Tree of Life (DToL) project. All raw sequence data and the assembly have been deposited in INSDC databases. Raw data and assembly accession identifiers are reported in Table 1.

## Author information

Members of the University of Oxford and Wytham Woods Genome Acquisition Lab are listed here: https://doi.org/10.5281/zenodo.4789928.

Members of the Darwin Tree of Life Barcoding collective are listed here: https://doi.org/10.5281/zenodo.4893703.

Members of the Wellcome Sanger Institute Tree of Life programme are listed here: https://doi.org/10.5281/zenodo.4783585.

Members of Wellcome Sanger Institute Scientific Operations: DNA Pipelines collective are listed here: https://doi.org/10.5281/zenodo.4790455.

Members of the Tree of Life Core Informatics collective are listed here: https://doi.org/10.5281/zenodo.5013541.

Members of the Darwin Tree of Life Consortium are listed here: https://doi.org/10.5281/zenodo.4783558.
