# Supplementary material for: The genome sequence of the Vapourer moth, Orgyia antiqua (Linnaeus, 1758)
Source: Wellcome Open Res. 2024 Oct 15;8:314. Originally published 2023 Jul 20. [Version 2] doi: 10.12688/wellcomeopenres.19480.2 (PMC11502997; doi:10.12688/wellcomeopenres.19480.2)
Supplement: Peer review history

# Reviewer report: Reviewer response for version 2

**DOI:** 10.21956/wellcomeopenres.25628.r106612

**Published:** 2024-11-12

**Authors:** Muzafar Riyaz

**Affiliations:** 1 Xavier Research Foundation, Loyola College of Education, Palayamkottai, Tamil Nadu, India

This study presents the genome sequence of *Orgyia antiqua* (Vapourer moth), detailing a 480.1 Mb assembly scaffolded into 14 chromosomal pseudomolecules and a mitochondrial genome of 15.4 kb. With high coverage from PacBio HiFi and 10X Genomics technologies, the assembly achieves a 98.8% BUSCO completeness and a QV of 62.1. The authors provide insights into the species' unique biological traits, such as female brachyptery and polyphagous larvae, which support its pest potential. Using Ensembl for annotation, they identified 12,475 protein-coding genes. Comprehensive protocols for DNA extraction, sequencing, and assembly are meticulously documented, and the genome data is openly accessible, supporting broader ecological and evolutionary research.

Are sufficient details of methods and materials provided to allow replication by others?

Yes

Is the rationale for creating the dataset(s) clearly described?

Yes

Are the datasets clearly presented in a useable and accessible format?

Yes

Are the protocols appropriate and is the work technically sound?

Yes

Reviewer Expertise:

Phylogenomics

I confirm that I have read this submission and believe that I have an appropriate level of expertise to confirm that it is of an acceptable scientific standard.

# Reviewer report: Reviewer response for version 2

**DOI:** 10.21956/wellcomeopenres.25628.r106589

**Published:** 2024-11-11

**Authors:** Yonghao Yu

**Affiliations:** 1 Plant Protection Research Institute, Guangxi Academy of Agricultural Sciences, Nanning, China

The authors present a genome of *Orgyia antiqua* assembly. The insect is a common lymantrin moth native to the European Palaearctic, but which has expanded into North America. It is widespread throughout a range of habitats wherever usable host plants are present, from parks and gardens to woodlands and scrublands. The work is meaningful. However, I think the genome sequence is not complete. It is still necessary to determine the location of the telomere and centromere sequences on each chromosome.

Are sufficient details of methods and materials provided to allow replication by others?

Yes

Is the rationale for creating the dataset(s) clearly described?

Yes

Are the datasets clearly presented in a useable and accessible format?

Yes

Are the protocols appropriate and is the work technically sound?

Partly

Reviewer Expertise:

Insect molecular ecology

I confirm that I have read this submission and believe that I have an appropriate level of expertise to confirm that it is of an acceptable scientific standard.

# Reviewer report: Reviewer response for version 2

**DOI:** 10.21956/wellcomeopenres.25628.r105123

**Published:** 2024-10-24

**Authors:** Will Nash

**Affiliations:** 1 Earlham Institute, Norwich, England, UK

I am most grateful to the authorship team for taking the time to address my comments. I think this improves the quality of the manuscript by increasing the repeatability of the methods presented. 

 I am happy to recommend this manuscript for indexing.

Are sufficient details of methods and materials provided to allow replication by others?

No

Is the rationale for creating the dataset(s) clearly described?

Yes

Are the datasets clearly presented in a useable and accessible format?

Yes

Are the protocols appropriate and is the work technically sound?

Yes

Reviewer Expertise:

Bioinformatics, Genomics, Assembly

I confirm that I have read this submission and believe that I have an appropriate level of expertise to confirm that it is of an acceptable scientific standard.

# Reviewer report: Reviewer response for version 1

**DOI:** 10.21956/wellcomeopenres.21578.r85719

**Published:** 2024-08-29

**Authors:** Will Nash

**Affiliations:** 1 Earlham Institute, Norwich, England, UK

Weir et al. present a chromosome level assembly and annotation of the genome of the Vapourer Moth ( *Orgyia antiqua*). The assembly is constructed from 54x PacBio HiFi data and 71x 10X read clouds, and scaffolded with Hi-C. The final assembly is 480.1 Mb over 14 chromosome scale scaffolds. One of these is assigned to the Z sex chromosome. The assembly is of excellent quality (QV: 62.1, BUSCO lepidoptera_odb10 98.9 % complete). >21k transcribed mRNAs are annotated.

 The introduction of the study is well written, interesting and cites diverse literature.

 The methods represent a cutting edge pipeline for genome assembly. The methods presented here are not consistent with other DToL genome papers and do not have references to established DToL protocols.io papers – is this because this assembly predates these? This needs to be clarified. https://wellcomeopenresearch.org/articles/9-46/v1 provides a better and more replicable set of methods. In line with my review of that study, I view the methods as described in the current manuscript as un-replicable: not enough information is provided about the Hi-C library preparation. This is a difficult and multistage process and needs more detail – this would allow readers to understand species or taxon specific modifications to Hi-C library preparation.

 The assembly and bioinformatic QC are all clearly described. I would like to see more detail on the specific settings used in each software – this would facilitate reproduction of the assembly and QC.

Are sufficient details of methods and materials provided to allow replication by others?

No

Is the rationale for creating the dataset(s) clearly described?

Yes

Are the datasets clearly presented in a useable and accessible format?

Yes

Are the protocols appropriate and is the work technically sound?

Yes

Reviewer Expertise:

Bioinformatics, Genomics, Assembly

I confirm that I have read this submission and believe that I have an appropriate level of expertise to state that I do not consider it to be of an acceptable scientific standard, for reasons outlined above.

# Response

**Published:** 2024-10-10

**Authors:** Tree of Life Team Sanger

**Affiliations:** Wellcome Trust Sanger Institute, UK

The reviewer correctly notes that this data note predates the publication of detailed methods protocols for the Wellcome Sanger Institute core laboratory. We have now included references to these protocols. Additionally, we have provided more detailed information on the Hi-C library preparation, including specifics of the preparation, library construction, and sequencing.

# Reviewer report: Reviewer response for version 1

**DOI:** 10.21956/wellcomeopenres.21578.r85720

**Published:** 2024-06-14

**Authors:** William Reid

**Affiliations:** 1 SUNY at Buffalo, Buffalo, New York, USA

In the data note "The genome sequence of the Vapourer moth, *Orgyia antiqua *(Linnaeus, 1758)", Weir and Boyes present an annotated chromosome-level assembly of *O. antiqua*. The sequencing, assembly, and genome annotation follow DToL protocols and resulted in a high-quality male genome for the moth. All data (raw and processed) are available with working links through ENA, NCBI, and rapid-release DToL in Ensembl. 

 A few minor suggestions are:

 In the background section, consider changing 'lymantrin' to Lymantrine to avoid confusion with the LdMNPV biocontrol product.

 Consider mentioning that the specimen used for RNA was male as well (although this should be obvious since it was collected through light trapping).

 Is it possible to put the link to the DToL site in Ensembl? (https://projects.ensembl.org/darwin-tree-of-life/ or ftp.ensembl.org/pub/rapid-release/species/Orgyia_antiqua/GCA_916999025.1)

Are sufficient details of methods and materials provided to allow replication by others?

Yes

Is the rationale for creating the dataset(s) clearly described?

Yes

Are the datasets clearly presented in a useable and accessible format?

Yes

Are the protocols appropriate and is the work technically sound?

Yes

Reviewer Expertise:

Insect transgenics

I confirm that I have read this submission and believe that I have an appropriate level of expertise to confirm that it is of an acceptable scientific standard.
